# Mapping SF-36 onto the EQ-5D index: how reliable is the relationship?

**DOI:** 10.1186/1477-7525-7-27

**Published:** 2009-03-31

**Authors:** Donna Rowen, John Brazier, Jennifer Roberts

**Affiliations:** 1 Health Economics and Decision Science, University of Sheffield, Regent Court, 30 Regent Street, Sheffield, S1 4DA, UK; 2 Department of Economics, University of Sheffield, 9 Mappin Street, Sheffield, S1 4DT, UK

## Abstract

**Background:**

Mapping from health status measures onto generic preference-based measures is becoming a common solution when health state utility values are not directly available for economic evaluation. However the accuracy and reliability of the models employed is largely untested, and there is little evidence of their suitability in patient datasets. This paper examines whether mapping approaches are reliable and accurate in terms of their predictions for a large and varied UK patient dataset.

**Methods:**

SF-36 dimension scores are mapped onto the EQ-5D index using a number of different model specifications. The predicted EQ-5D scores for subsets of the sample are compared across inpatient and outpatient settings and medical conditions. This paper compares the results to those obtained from existing mapping functions.

**Results:**

The model including SF-36 dimensions, squared and interaction terms estimated using random effects GLS has the most accurate predictions of all models estimated here and existing mapping functions as indicated by MAE (0.127) and MSE (0.030). Mean absolute error in predictions by EQ-5D utility range increases with severity for our models (0.085 to 0.34) and for existing mapping functions (0.123 to 0.272).

**Conclusion:**

Our results suggest that models mapping the SF-36 onto the EQ-5D have similar predictions across inpatient and outpatient setting and medical conditions. However, the models overpredict for more severe EQ-5D states; this problem is also present in the existing mapping functions.

## Background

Clinical trials use a multitude of health status measures in order to measure health and health related quality of life. However, most of these measures cannot be used in assessments of cost effectiveness using cost per Quality Adjusted Life Year (QALY). Preference-based measures such as the EQ-5D are commonly used to do this, but are not always used in clinical studies. One solution to this problem is to apply a mapping function to convert non-preference based health data into one of the generic preference-based measures; this is helpful to those submitting evidence to agencies such as NICE [1]. However the accuracy and reliability of the mapping models employed is largely untested, and there is little evidence of their suitability in patient datasets.

A recent review of mapping non-preference-based measures onto generic preference-based measures [2] found 29 studies. However, most of these used simple OLS modelling procedures on comparatively small data sets. Further, existing studies have neglected to investigate the robustness of the models across patient data sets.

The purpose of this paper is to examine whether mapping models are reliable and accurate in terms of their predictions for a large and varied patient dataset. The mapping relationship examined here is between the EQ-5D index, a generic preference-based measure of health related quality of life and the SF-36, a generic non-preference-based health status measure commonly used in clinical trials. A mapping relationship is estimated using a range of techniques and statistical specifications. We examine the mapping relationship across inpatient and outpatient settings and medical conditions according to ICD classification. Furthermore, we compare the mapping approach used here to existing models [3,4] in terms of predictive performance.

## Methods

### The model

The SF-36 assesses health across eight dimensions using 36 items. The SF-36 produces a score on a 0–100 scale for each of the eight dimensions, which are specific health domains such as physical functioning, social functioning and vitality. These scores are not comparable across dimensions and are not based on individual preferences, therefore they cannot be used to generate QALYs. The SF-36 can be used to generate a preference-based index via the SF-6D [5].

The EQ-5D is the most widely used generic preference-based measure of health-related quality of life which produces utility scores anchored at 0 for dead and 1 for perfect health. The utility scores represent preferences for particular health states. The descriptive system has 5 dimensions (mobility, self-care, usual activity, pain/discomfort and anxiety/depression) and 3 levels (no problems, some problems, extreme problems) which create 243 unique health states. This study uses the UK TTO value set in its main analysis [6]. The EQ-5D valued using the UK TTO value set is preferred by NICE [1]. The SF-6D has been found to differ from the EQ-5D [7] and so to achieve comparability between studies using different measures this paper explores an alternative strategy of mapping.

### Model specifications

Regression analysis is used to examine the relationship between the EQ-5D utility score and the SF-36 using the 8 dimension scores; physical functioning, role-physical, bodily pain, general health, vitality, social functioning, role-emotional and mental health, squared dimension scores and interaction terms derived using the product of two dimension scores. The dependent variable, the EQ-5D utility score, is measured on a -1 to 1 scale. The 8 dimension scores of the SF-36 are rescaled onto a 0–1 scale to enable easier interpretation of the results and the squared terms and interaction terms are generated using the rescaled scores.

Three models are estimated: (1) all dimensions; (2) all dimensions and squared terms; (3) all dimensions, squared terms and interactions. The general model is defined as

(1)

where *i *= 1,2,..., *n *represents individual respondents and *j *= 1,2,..., *m *represents the 8 different dimensions. The dependent variable, *y*, represents the EQ-5D utility score, **x **represents the vector of SF-36 dimensions, **r **represents the vector of squared terms, **z **represents the vector of interaction terms and *ε*_*ij *_represents the error term. This is an additive model which imposes no restrictions on the relationship between dimensions. The squared terms are designed to pick up non-linearities in the relationship between dimension scores and the EQ-5D index. There is no reason for it to be linear and there is evidence in physical functioning, for example, that the same differences in scores at the lower end of the scale indicate larger differences in functioning than at the upper end [8]. Interaction terms are important since there is evidence from other measures that dimensions are not additive [9]. Statistical measures of explanatory power, predictive ability, and model specification are reported.

The sample used here is a patient dataset (described below) where respondents are included each time they are treated, and hence some respondents have multiple observations. Random effects models are used to take account of this data structure. The estimated models are used to generate predicted EQ-5D scores. Predictive ability is assessed using line graphs of the observed and predicted EQ-5D utility scores ordered by observed tariff value of EQ-5D state, mean error, mean absolute error and mean squared error.

EQ-5D utility scores are known to exhibit a ceiling effect, where a large proportion of subjects rate themselves in full health with a utility score of 1, and hence the data can be interpreted as being bounded or censored at 1. Ignoring the bounded nature of the EQ-5D will result in biased and inconsistent estimates, and hence the random effects tobit model is an appropriate alternative [10]. The tobit model with an upper censoring limit of 1 is defined as



(2)

where  is the observed EQ-5D utility score and *y*_*i *_is the bounded measure of the EQ-5D score.

However, the tobit model also produces biased estimates in the presence of heteroscedasticity or non-normality [10,11]. The censored least absolute deviations (CLAD) model is also used here since it produces consistent estimates in the presence of heteroscedasticity and non-normality [10,12]. STATA version 9 was used for all regression analysis and CLAD was performed using programs written for [13], SPSS version 12 was used for statistical analysis.

### Reliability and robustness

In order to examine whether the estimated relationships are reliable and robust across inpatient and outpatient setting and medical conditions, we estimate model (3) as outlined above for subsets of the sample data^i^. The model is estimated for inpatients and outpatients and for the medical conditions of neoplasms, diseases of the circulatory system and diseases of the digestive system as measured according to ICD classifications C, I and K respectively.

### Comparison to existing mapping functions

Our models are compared to existing approaches [3,4,10] to determine whether their mapping approaches are more or less reliable for a patient dataset. The existing models from the literature are estimated using the published results and algorithms rather than re-estimating the models using our dataset. We take this approach because mapping is used in economic evaluations to estimate the EQ-5D using the SF-36 (or SF-12) when this is the only health status measure that has been included in the trial. Therefore in practical applications the published results and algorithms are used and it is not feasible to re-estimate the model.

Franks et al. [3] regress the EQ-5D utility score on PCS-12 and MCS-12, squared terms and cross-products using OLS. PCS and MCS are the physical and mental component summary scores estimated using factor analysis and shown to contain most of the information contained in the 8 dimensions of the SF-36 [14]. In accordance with this approach PCS-12 and MCS-12 are centred on the means used in the paper [3] and the published coefficients are used to produce predicted EQ-5D utility scores.^ii ^Another study [15] uses similar variables and estimation techniques to [3] in order to predict EQ-5D scores from the SF-12 and hence the model is not analysed here separately.

Gray et al. [4] use a response mapping approach that uses a multinomial logit model to estimate the probability that a respondent will choose a particular level for each dimension of the EQ-5D using responses to the 12 items included in the SF-12 (general health, climbing stairs, moderate activities, accomplish less due to physical health, work limitations, accomplish less due to emotional problems, work carefully, pain interference, calm, energy, down-hearted and low, interference with social activities). Subsequently predicted EQ-5D level responses for each dimension are generated using Monte Carlo simulation methods and the corresponding EQ-5D utility score for that health state is calculated. We use the available algorithm to predict EQ-5D utility scores [4].^iii^

Sullivan and Ghushchyan [10] regress the US EQ-5D utility score on PCS-12 and MCS-12, the product of PCS-12 and MCS-12 and sociodemographic variables using OLS, tobit and CLAD. It is not appropriate to use the exact model [10] as they use the US-based EQ-5D values [16] rather than the UK-based values [6] and further only report models including sociodemographic variables unavailable in our dataset. Instead we have used the tobit and CLAD estimation techniques suggested in [10] as outlined above and re-estimated the model using our dataset.

### The data

The Health Outcomes Data Repository, HODaR, is a dataset collated by Cardiff Research Consortium. The data is collected from a prospective survey of inpatients and outpatients at Cardiff and Vale NHS Hospitals Trust, which is a large University hospital in South Wales, UK. The survey is linked to existing routine hospital health data to provide a dataset with sociodemographic, health related quality of life and ICD classification data^iv^. The survey includes all subjects aged 18 years or older and excludes individuals who are known to have died. The survey also excludes people with a primary diagnosis on admission of a psychological illness or learning disability. As well as information on inpatients, the survey includes outpatient clinics on a rotational basis where all patients within the selected clinic are surveyed. The response rate in HODaR prior to October 2003 was around 36% and subsequently strategies were implemented to improve response rates to around 50% [17].

The inpatient sample has 31,236 eligible observations across 27,620 individuals from August 2002 to November 2004, and of these there are 25,783 complete responses across 23,179 individuals for SF-36 and EQ-5D questions and hence this is the sample used here. The outpatient sample has 9,081 eligible observations across 8,610 individuals collected from June 2002 to November 2004, and of these there are 7,465 complete responses across 7,122 individuals. The dataset covers a wider range of conditions and severity than the general population datasets used in existing mapping approaches, and hence may be more similar to datasets used in economic evaluation.

## Results

Table 1 provides descriptive statistics on health status. The inpatient and outpatient samples in the HODaR dataset demonstrate substantial health problems according to the EQ-5D, the SF-36 dimension scores and the SF-12 summary scores in comparison to UK population norms [18,19]. Health appears similar between inpatients and outpatients. In comparison to the inpatient sample the outpatient sample has a larger proportion of females and a lower mean age.

**Table 1:** Descriptive data for the inpatient and outpatient samples

	Inpatients			Outpatients			UK population norms^v^
	Mean (SD)	Median	Inter-quartile range	Mean (SD)	Median	Inter-quartile range	Mean (SD)

EQ-5D index	0.68(0.31)	0.73	0.413	0.69(0.31)	0.73	0.38	0.86(0.23)
							
*SF-36 dimension scores*							
Physical functioning	58.90(33.53)	65.00	60.00	62.29(33.39)	70.00	60.00	88.40(17.98)
Social functioning	63.43(33.16)	66.67	66.67	66.35(32.02)	77.78	55.56	88.01(19.58)
Role physical	28.74(41.90)	0.00	75.00	34.21(44.11)	0.00	100.00	85.82(29.93)
Role-emotional	51.14(47.14)	66.67	100.00	54.32(46.99)	66.67	100.00	82.93(31.76)
Mental health	69.54(23.13)	76.00	32.00	69.58(22.54)	76.00	32.00	73.77(17.24)
Vitality	45.36(25.73)	45.00	40.00	45.60(25.37)	45.00	40.00	61.13(19.67)
Bodily pain	58.13(28.68)	55.56	44.44	58.86(28.84)	55.56	55.56	81.49(21.69)
General health	52.80(26.28)	52.00	47.00	53.29(25.91)	52.00	47.00	73.52(19.90)
							
*SF-12 summary scores*							
Physical component score	38.25(12.18)	36.68	21.49	39.51(12.34)	38.47	22.50	50.00(10.00)
Mental component score	44.85(11.69)	46.21	19.38	45.03(11.45)	46.92	19.07	50.00(10.00)
							
Mean age	58.14			55.55			
Female	52%			61%			
							
*N*	25,783			7,465			

### Inpatients

Table 2 shows the results of the regression analyses using dimensions, squared terms and interaction terms for the inpatient dataset. The results show that all dimensions are always significant with the exception of role physical, vitality and role emotional and are positive with the exception of role physical and vitality. The results indicate that the squared terms for physical functioning, bodily pain, social functioning and mental health are always significant and negative and many interaction terms are also significant with mixed signs. Statistical measures reported in Table 2 of within, between and overall R-squared, root mean squared error, rho and Wald chi-squared indicate that models (2) and (3) perform better than model (1).

**Table 2:** Prediction models for inpatients using dimensions, squared terms and interaction terms

	Random effects GLS			Tobit	CLAD
	(1)	(2)	(3)	(4)	(5)

*Dimensions*					
Physical functioning (PF)	0.332*	0.548*	0.559*	0.559*	0.663*
Role physical (RP)	-0.060*	-0.021	-0.146*	-0.146*	-0.475*
Bodily pain (BP)	0.303*	0.747*	0.715*	0.713*	0.733*
General health (GH)	0.169*	0.322*	0.407*	0.407*	0.325*
Vitality (VIT)	-0.039*	0.007	0.017	0.017	-0.142*
Social functioning (SF)	0.115*	0.256*	0.293*	0.293*	0.525*
Role-emotional (RE)	0.010*	0.014	0.067*	0.067*	-0.024
Mental health (MH)	0.237*	0.577*	0.483*	0.483*	0.527*
					
*Dimensions squared*					
Physical functioning (PF)		-0.250*	-0.227*	-0.227*	-0.082*
Role physical (RP)		0.043*	0.001	0.001	-0.056*
Bodily pain (BP)		-0.378*	-0.330*	-0.329*	-0.171*
General health (GH)		-0.137*	0.032	0.031	0.167*
Vitality (VIT)		-0.014	-0.012	-0.012	0.063
Social functioning (SF)		-0.179*	-0.163*	-0.163*	-0.182*
Role-emotional (RE)		0.017	0.034	0.034	0.058*
Mental health (MH)		-0.321*	-0.242*	-0.242*	-0.152*
					
*Interaction terms*					
PF × RP			0.022	0.022	0.185*
PF × BP			-0.032	-0.031	-0.192*
PF × GH			0.073	0.073	-0.009
PF × VIT			-0.132*	-0.132*	-0.078
PF × SF			-0.023	-0.023	-0.246*
PF × RE			0.047*	0.047*	0.045*
PF × MH			-0.014	-0.013	-0.054
RP × BP			0.019	0.019	0.097*
RP × GH			0.068*	0.068*	0.215*
RP × VIT			0.050	0.049	0.031
RP × SF			0.067*	0.067*	0.108*
RP × RE			-0.012	-0.012	0.013
RP × MH			0.022	0.022	0.154*
BP × GH			-0.217*	-0.217*	-0.208*
BP × VIT			-0.002	-0.002	0.120*
BP × SF			0.055	0.055	-0.070*
BP × RE			-0.038	-0.038	0.039*
BP × MH			0.131*	0.131*	-0.075
GH × VIT			-0.066	-0.066	-0.200*
GH × SF			-0.157*	-0.158*	-0.144*
GH × RE			-0.033	-0.033	-0.019
GH × MH			-0.084	-0.084	-0.114*
VIT × SF			0.143*	0.143*	0.174*
VIT × RE			-0.020	-0.019	-0.021
VIT × MH			0.023	0.022	0.095
SF × RE			-0.023	-0.023	-0.024
SF × MH			-0.065	-0.065	-0.133*
RE × MH			-0.048	-0.048	-0.035
Constant	0.0071	-0.2493*	-0.256*	-0.256*	-0.289*
					
Within R-squared	0.18	0.21	0.22	-	-
Between R-squared	0.67	0.70	0.71	-	-
Overall R-squared	0.67	0.70	0.71	-	-
Root MSE	0.15	0.15	0.15	-	-
Rho	0.28	0.24	0.24		
Wald Chi-squared	48380.12	56129.39	57195.96		

Note: * significant at 1%

**Figure 1:**
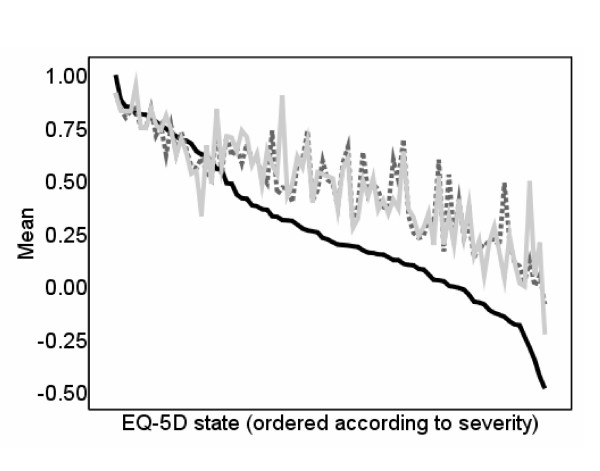
**Observed and predicted EQ-5D scores: Inpatients and outpatients random effects GLS model**.  EQ-5D score  Inpatient predictions  Outpatient predictions

Table 3 reports mean error, mean absolute error (MAE) and mean squared error (MSE) of predicted compared to actual utility scores by EQ-5D utility range for all models estimated in Table 2. Table 3 indicates that the estimation techniques of tobit and CLAD do not clearly improve the accuracy of the generated predictions as MAE and MSE are not reduced. Model (3) estimated using random effects GLS have the most accurate predictions as indicated by MAE and MSE. Figure 1 and MAE and MSE reported in table 3 suggest that the model predicts well for milder health states, but overpredicts the value of more severe EQ-5D states. All models estimated in Table 2 suffer from the same problem.

**Table 3:** Mean error, mean absolute error and mean squared error of predicted compared to actual utility scores by EQ-5D utility range for random effects GLS models, random effects tobit models, CLAD model, Franks et al. model and Gray et al. model

*EQ-5D utility score*	*Random effects GLS*			*Random effects tobit*	*CLAD*	*Franks et al*. [3]	*Gray et al*. [4]
	*(1)*	*(2)*	*(3)*	*(4)*	*(5)*		

*Mean error*							
<0	-0.340	-0.266	-0.260	-0.260	-0.269	-0.252	-0.213
0–0.249	-0.241	-0.219	-0.217	-0.216	-0.237	-0.144	-0.144
0.25–0.499	-0.191	-0.189	-0.191	-0.182	-0.219	-0.064	-0.081
0.5–0.6.99	0.098	0.072	0.070	0.081	0.052	0.201	0.135
0.7–0.799	-0.004	-0.024	-0.024	0.023	-0.044	0.095	0.056
0.8–0.899	0.041	0.034	0.034	0.089	0.004	0.167	0.114
0.9–1.0	0.064	0.086	0.085	0.178	0.025	0.154	0.123
Full index	-0.001	0.000	0.000	0.041	-0.031	0.101	0.059
							
*Mean absolute error*							
<0	0.340	0.271	0.266	0.266	0.278	0.254	0.272
0–0.249	0.244	0.238	0.238	0.236	0.260	0.175	0.278
0.25–0.499	0.202	0.215	0.219	0.210	0.247	0.136	0.282
0.5–0.699	0.138	0.131	0.130	0.123	0.122	0.211	0.210
0.7–0.799	0.105	0.098	0.095	0.063	0.102	0.147	0.145
0.8–0.899	0.106	0.088	0.085	0.089	0.092	0.183	0.172
0.9–1.0	0.086	0.086	0.085	0.178	0.092	0.154	0.123
Full index	0.138	0.129	0.127	0.142	0.133	0.178	0.186
							
*Mean squared error*							
<0	0.132	0.099	0.097	0.097	0.110	0.082	0.135
0–0.249	0.078	0.080	0.080	0.078	0.095	0.048	0.123
0.25–0.499	0.061	0.066	0.067	0.060	0.085	0.032	0.102
0.5–0.699	0.028	0.028	0.028	0.026	0.026	0.060	0.094
0.7–0.799	0.017	0.015	0.014	0.009	0.018	0.034	0.052
0.8–0.899	0.019	0.015	0.014	0.015	0.016	0.051	0.065
0.9–1.0	0.015	0.013	0.013	0.034	0.013	0.037	0.042
Full index	0.033	0.030	0.030	0.033	0.033	0.048	0.076

### Inpatients and outpatients

Figure 1 shows the observed and predicted EQ-5D scores for inpatients and outpatients, ordered by observed tariff value of the EQ-5D state. The predictions are generated using model (3) estimated using random effects GLS. The mapping relationship follows the same pattern across inpatient and outpatient settings and both overpredict for more severe EQ-5D states. Wald test statistics calculated to determine whether the estimated coefficients for inpatients are equal to the estimated coefficients for outpatients for models with exactly the same specification indicate that the estimated coefficients are not equal and hence the models are not robust to different samples. However, differences in predictions are small with mean absolute difference at the state level of 0.069 and mean squared difference of 0.012. Wald test statistics were also calculated for subsets of the inpatient sample according to medical condition for the ICD classifications with the largest number of observations in the dataset, which are the medical conditions of neoplasms (n = 2,574), diseases of the circulatory system (n = 3,522) and diseases of the digestive system (n = 3,114) as measured according to ICD classifications C, I and K respectively. The test statistics again indicate that the estimated coefficients are not equal and hence are not robust across subsets of the inpatient sample according to medical condition, but differences in predictions are small with highest mean absolute difference at the state level of 0.054 and highest mean squared error of 0.005.

### Comparison to existing mapping

**Figure 2:**
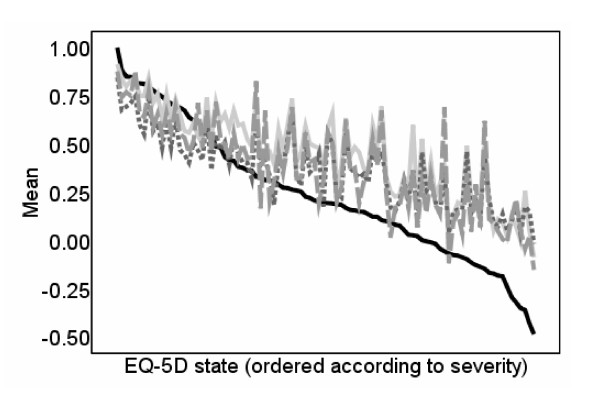
**Observed and predicted EQ-5D scores: Comparison to existing mapping functions**.  EQ-5D score  Predictions using our model  Franks et al. [3] predictions  Gray et al. [4] predictions

Figure 2 shows observed and predicted EQ-5D utility scores for model (3) and for existing approaches [3,4]. The mapping relationship is similar across all approaches and they all overpredict for more severe EQ-5D states. Table 3 shows mean error, mean absolute error and mean square error of predicted compared to actual utility scores by EQ-5D utility range for existing approaches [3,4]. As indicated by Figure 2, the errors are higher for more severe health states for all models. Our model performs better than the existing models as reported by mean error, mean absolute error and mean square error.

### Re-estimation of the EQ-5D

**Figure 3:**
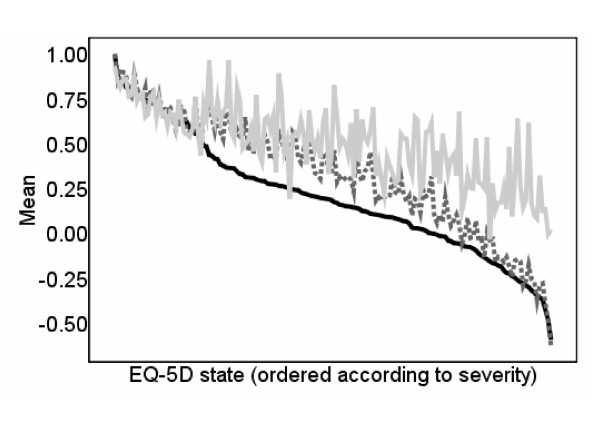
**Observed and predicted EQ-5D scores: Using EQ-5D tariff re-estimated without an N3 term using the MVH data**.  EQ-5D score  Reestimated EQ-5D score  Predictions using reestimated EQ-5D score

One hypothesis is that the predictions may be poor for more severe EQ-5D states because they all have at least one dimension at the most severe level and the EQ-5D model uses an 'N3' term, a dummy variable for states with at least one dimension at the most severe level. The 'N3' term was used in the original UK modelling [6], but has not been included in all the models of other EQ-5D valuation studies (see for example the US valuation study, [16]). The inclusion of the N3 term may be a reason why the utility score is overpredicted for the more severe states which have at least one dimension at the most severe level. We re-estimated the EQ-5D tariff without the N3 term using the same data and methods as the original UK tariff [6]. The re-estimated tariff and the original UK tariff [6] produce similar scores for mild and very severe health states but deviate for more moderate health states, with mean difference in tariff values at the state level of 0.134 and mean squared difference of 0.026. Figure 3 plots the observed and predicted EQ-5D utility scores using a re-estimated version of the EQ-5D and plots this alongside the UK tariff values [6]. The predicted values for the re-estimated EQ-5D scores still overpredict for more severe states, but not as much as previously, with MAE of 0.106 and MSE of 0.021 in comparison to MAE of 0.127 and MSE of 0.030 for the predictions based on the UK tariff [6]. However the PITS state is overpredicted by 0.63 for the re-estimated EQ-5D scores and 0.61 for the predictions based on the UK tariff [6].

### US-based EQ-5D

**Figure 4:**
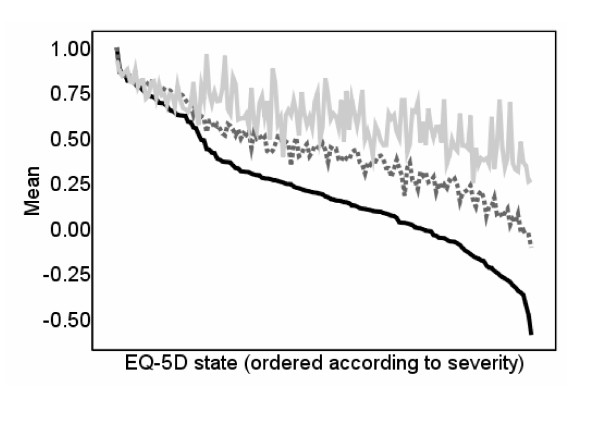
**Observed and predicted EQ-5D scores: Using the US-based EQ-5D tariff**.  EQ-5D score  US-based tariff EQ-5D score  Predictions using US-based tariff

The re-estimated UK tariff and the UK tariff [6] produce similar scores for mild and very severe health states and hence the preferences regarding more severe health states may be a property of the dataset rather than the estimation technique used for the valuation. The US-based EQ-5D tariff has a smaller range from 1 to -0.11 and hence has higher scores for very severe states, suggesting that the mapping relationship between the US-based EQ-5D index and the SF-36 may not suffer from overprediction for more severe health states. Figure 4 plots the observed and predicted EQ-5D scores using the US-based tariff values [16] alongside the UK tariff values [6]. This demonstrates that the predicted values for the US-based EQ-5D values still overpredict for more severe states, but the estimates are more reliable than those plotted in figure 3 with MAE of 0.110 and MSE of 0.022 in comparison to MAE of 0.127 and MSE of 0.030 for the predictions based on UK tariff [6]. The PITS state is overpredicted by 0.38 for the US-based EQ-5D values and 0.86 for the predictions based on UK tariff [6].

## Discussion

The patient dataset used here is much better than general population datasets in terms of diversity of conditions and severity of health. Our results suggest that the mapping relationship between the EQ-5D index and the SF-36 for a large and varied UK patient dataset is reliable and accurate across inpatient and outpatient settings and medical conditions. One advantage of using this approach in the UK is that the EQ-5D is currently recommended by NICE (2008) for use in economic evaluation. NICE (2008) also state that mapping can be used when EQ-5D was not included in the trial. However, our results indicate that the mapping relationship is not accurate and reliable for more severe EQ-5D health states. The inclusion of squared and interaction terms in the models improves diagnostics, mean error, MAE and MSE, suggesting that the mapping relationship is non-linear and dimensions are additive. The mapping approach used here is compared to existing approaches [3,4] and all suffer from overprediction for more severe EQ-5D health states. The added complexity of the response mapping approach used by Gray et al. [4] does not seem to improve the predictability for all health states in comparison to our approach.

One potential reason for the overprediction for more severe health states are the floor effects of the SF-36. We have tried to account for these floor effects by using squared terms and interaction terms in our model, but, as the figures illustrate, this does not resolve the problem. We also tried re-estimating the EQ-5D utility tariff using the original dataset used to estimate the UK tariff [6] but omitting the N3 term. Although Figure 3 demonstrates better predictions for more severe health states, the problem of overprediction is still evident. Indeed, if the preferences regarding more severe health states is a property of the dataset rather than the estimation technique, then the valuation produced here will still demonstrate the same properties. We also estimated our model using the US-based EQ-5D values, and although Figure 4 demonstrates better predictions for more severe health states, again the problem of overprediction is still evident.

The importance of the problem of overprediction in economic evaluations is difficult to measure, since it depends on the patient group and the effect of treatments. Ara and Brazier [20] predict mean cohort EQ-5D utility values using mean cohort scores for the dimensions of the SF-36 from published datasets. They find mean errors of 0.285 and 0.158 in prediction for the 5 out of 63 cohorts in an out of sample dataset with mean EQ-5D utility value below 0.175 and between 0.175 and 0.35 respectively. The impact at the group level may be less important since few patients have EQ-5D utility values below 0.5, and the inpatient and outpatient datasets used here each have 17% of observations with an EQ-5D utility value below 0.5, suggesting that not many observations will be affected by the overprediction for more severe states that is presented here. Therefore for most studies this may not matter, only where many patients have EQ-5D utility values below 0.5.

The results suggest that there are differences in the EQ-5D and SF-36 health status measures for more severe health states which make mapping unreliable for these states. Another finding is that the vitality, role physical and role-emotional dimensions of the SF-36 did not significantly effect the EQ-5D index, hence interventions aimed at improving these dimensions will not be reflected in the mapping model. However, these domains were found to be important to members of the public in the valuation of the SF-6D [5]. Mapping is increasingly being used between condition specific measures and generic measures of health (refer to [2]). However, the lack of overlap in the dimensions covered by many condition specific measures and EQ-5D limit the usefulness of this approach as these problems may be worsened if the health domains included in the measures are different.

## Conclusion

Mapping enables utility scores to be estimated in trials where a non-preference based health status measure has been used but no generic preference-based measure. Our results suggest that approaches mapping the SF-36 onto the EQ-5D are robust across setting and medical condition but overpredict for more severe EQ-5D states. Our results raise doubt over the suitability of mapping for patient datasets which have a proportion of subjects with poorer health or where dimensions are not represented in the target measure. Potential policy implications are that mapping the SF-36 onto the EQ-5D can be useful, but may not be suitable for all populations.

## Competing interests

The authors declare that they have no competing interests.

## Authors' contributions

JB and JR conceived the research question and provided technical expertise for the study. DR undertook the data analysis and wrote the manuscript. All authors contributed to the writing of the manuscript and read and approved the final manuscript.

## Note

^i ^The estimation results are not reported here but are available from the authors.

^ii ^Other models are estimated in [3] but these are not analysed here as these models use demographic variables not available in the dataset used here. Furthermore it was found that more complex models explained only minimally additional variance [3].

^iii ^The algorithm is available from the HERC website 

^iv ^See [17] for further details on HODaR.

^v ^EQ-5D population norms obtained from [18] for the Measurement and Valuation of Health survey and SF-36 population norms obtained from [19] for the Oxford Healthy Life Survey.
